# Combined Maternal and Paternal Low‐Dose N‐Nitrosodimethylamine Exposure: Maternal Alterations and Developmental Toxicity in Rats

**DOI:** 10.1002/bdr2.2540

**Published:** 2025-10-22

**Authors:** Julia Tavares Alves, Luara Magalhães, Julia Vitoria Francisco de Aquino, Otávio Galassi Vitale, Giovanna Ferrari Rosalem, Giovanna Kugel Marraschi, Reggina Lorena Mellado Castro, Sara Tawany Caetano dos Santos, Livia Trippe Nagaoka, Julia Stein, Hamilton Hisano, Bárbara Campos Jorge, Arielle Cristina Arena

**Affiliations:** ^1^ Department of Structural and Functional Biology Institute of Biosciences of Botucatu, Universidade Estadual Paulista—UNESP Botucatu São Paulo Brazil; ^2^ Embrapa Environment – Jaguariúna Jaguariúna São Paulo Brazil; ^3^ Toxicological Information and Assistance Center (CIATOX), Institute of Biosciences of Botucatu, Univ. Estadual Paulista—Botucatu (UNESP) Botucatu São Paulo Brazil

**Keywords:** embryotoxicity, environmental contaminants, fetal development, nitrosamines, skeletal anomalies

## Abstract

**Background:**

N‐Nitrosodimethylamine (NDMA) is an organic xenobiotic compound and a well‐established mutagen and carcinogen. Its potential to cause reproductive and developmental toxicity at low, relevant doses is unclear. This study evaluated the effects of combined maternal and paternal NDMA exposure, both preconceptionally and during gestation, on maternal health and embryotoxicity in rats.

**Methods:**

Rats were treated orally with vehicle (5 males, 10 females) or NDMA (7.2 ng/kg/day; 5 males, 12 females). Both sexes were exposed from postnatal day (PND) 60 to 90 (31 days); females continued during mating, and pregnant females from gestational day (GD) 0 to 20. On GD 20, dams were euthanized for hemato‐biochemical and embryofetal evaluations.

**Results:**

Clinical toxicity, fertility indices, and embryofetal parameters did not differ between groups. In NDMA‐exposed dams, hematological analyses showed a significant reduction in lymphocyte percentages, an increase in monocyte counts, and a decrease in basophils, accompanied by elevated serum urea levels and reduced alanine aminotransferase (ALT) activity. Placental analyses were comparable between groups. Female fetuses exhibited a reduction in appropriate for gestational age (AGA) classifications with a concomitant increase in large for gestational age (LGA), while in males a borderline increase in placental efficiency was detected. No significant external, visceral, or encephalic malformations were observed. However, skeletal analysis revealed a significantly increased incidence of supernumerary ribs in NDMA‐exposed fetuses.

**Conclusion:**

Combined maternal and paternal exposure to low‐dose NDMA produced measurable adverse effects on maternal health and specific aspects of fetal development, highlighting its potential risk even at environmentally relevant exposure levels.

## 1 Introduction

N‐nitrosamines are environmental contaminants of increasing public health concern due to their widespread presence in drinking water, food, and industrial environments (Gushgari and Halden 2018). These compounds can form unintentionally during industrial processes involving nitrites or nitrates and amines and are commonly generated in settings such as tanneries, fish‐processing facilities, foundries, pesticide and dye manufacturing sites, and the rubber and tire industries (US‐EPA 2016). Environmentally, nitrosamines are frequently detected in drinking water and occur in smoked and grilled foods, dairy products, and certain vegetables (ANVISA 2021). Their broad distribution results in widespread human exposure and underscores the need for further research into their potential health effects.

Among nitrosamines, N‐nitrosodimethylamine (NDMA) is one of the most prevalent and has been classified as probably carcinogenic to humans (Group 2A) by the International Agency for Research on Cancer (IARC 2010). This classification is qualitative and not based on defined threshold concentration. However, regulatory agencies have established benchmark values for risk assessment. For example, the US EPA calculated an oral cancer slope factor of 51 (mg/kg·day)^−1^, corresponding to a lifetime excess cancer risk of 1 × 10^−5^ at an intake of approximately 0.2 ng/kg·day (U.S. EPA 2002). Similarly, Health Canada set a maximum acceptable concentration of 40 ng/L for NDMA in drinking water, associated with a lifetime cancer risk of 10^−5^ (Health Canada 2011). Concerns regarding NDMA exposure have further intensified due to its detection in several widely used pharmaceutical products, including valsartan, irbesartan, losartan, metformin, ranitidine, and nizatidine (Paustenbach et al. 2024).

In addition to these cancer risk benchmarks, dietary intake represents a major route of human exposure to nitrosamines, with NDMA concentrations varying widely depending on the food source. Daily NDMA intake ranges from 0.0004 to 0.23 μg in cured meats, 0.0004 to 1.02 μg in smoked meats, and 0.0006 to 0.13 μg in grilled meats (FDA 2018; Snodin and Elder 2019). Global estimates of average per capita NDMA intake include 251 ng/day in China, 136 ng/day in the United States, 87.6 ng/day in Canada, and 188 ng/day in France (Li et al. 2021). In Brazil, NDMA has been detected in the São Paulo metropolitan area's drinking water, with a detection frequency of 89% and an average concentration of 67 ng/L (Vizioli et al. 2021). Complementing the slope factor and drinking water guidelines, regulatory agencies have also proposed acceptable daily intake (ADI) values, currently set at 96 ng/day for NDMA and 26 ng/day for nitrosodiethylamine (NDEA) (ANVISA 2021; FDA 2020).

NDMA is recognized as a potent mutagen and hepatotoxin in rodents (Johnson et al. 2021; Peto et al. 1984), with evidence supporting its transplacental carcinogenicity, particularly during late gestation (Anderson et al. 1989; Huang and Catalano 1994). NDMA exhibits clastogenic activity, inducing DNA damage through formaldehyde formation and reactive oxygen species (ROS) generation (ATSDR 2022). Although studies on the developmental and reproductive toxicity of nitrosamines are limited, both epidemiological and animal research have reported adverse outcomes, including reduced fetal body weight and increased fetal mortality following gestational NDMA exposure. These effects were observed after oral gavage administration of 15 or 20 mg/kg NDMA, as well as after intraperitoneal treatment with 0.1 mmol (7.4 mg/kg) NDMA (Anderson et al. 1989; ATSDR 2022; Napalkov and Alexandrov 1968; Nishie 1983). This may be due to the accumulation of nitrosamines in the placenta, which can damage both maternal and fetal DNA. For instance, NDMA exposure during pregnancy significantly increased levels of 8‐oxo‐2′‐deoxyguanosine, a marker of oxidative DNA damage, in maternal, placental, and fetal tissues, suggesting potential long‐term effects on offspring (Sipowicz et al. 1997; Zhao et al. 2023). NDMA is also capable of crossing the placental barrier and reaching the fetal circulation, thereby directly exposing the developing fetus (Annola et al. 2009).

In addition to maternal susceptibility, paternal exposure to NDMA may also influence reproductive outcomes. Reactive metabolites of NDMA can reach the testes and cause DNA alkylation and oxidative damage in germ cells, leading to impaired sperm quality and the potential transmission of genetic and epigenetic alterations to the offspring. Experimental evidence has shown that paternal exposure to nitrosamines may reduce reproductive efficiency and compromise embryonic development (Liu et al. 2025; Pang et al. 2023). These findings highlight the importance of evaluating combined parental exposures to capture the full spectrum of potential reproductive risks.

Despite these toxicological findings, the effects of low‐level, regulatory‐relevant NDMA exposures on reproductive and developmental outcomes remain poorly characterized. This knowledge gap is especially concerning because early life stages—such as embryogenesis and fetal development—are highly sensitive to environmental toxicants due to rapid cell division and dynamic epigenetic processes (Gore et al. 2015; Ho et al. 2017). Although NDMA induces pronounced toxicity in rodents following high‐dose oral (20–40 mg/kg) or inhalation exposure (5–10 ppm) (ATSDR 2019), its effects under chronic, low‐dose conditions—defined as exposures at or near regulatory benchmarks, such as the human ADI of 96 ng/day (≈2 ng/kg/day) and the corresponding rat‐equivalent dose of 7.2 ng/kg/day used in this study—remain inadequately understood.

Therefore, this study aims to evaluate the effects of combined maternal and paternal exposure to NDMA—both prior to conception and throughout gestation—on maternal toxicity and embryo‐fetal development in a rat model. These findings may provide critical insights into the reproductive and developmental risks associated with environmentally relevant NDMA exposure, thereby informing regulatory decisions and public health interventions.

## 2 Materials and Methods

### 2.1 Chemical and Dosage

N‐Nitrosodimethylamine (N7756; purity 99.0%) was obtained from Sigma‐Aldrich. The dose selected was based on the calculation of the acceptable daily intake for humans (considering an adult with a body weight of 50 kg) of 96 ng/day (ANVISA 2021; FDA 2021), equivalent to approximately 2 ng/kg/day. To account for interspecies differences, the human‐to‐rat dose conversion formula was applied: Human dose = Rat dose × (Rat weight/Human weight)^0.25^ (US‐EPA 2011). Considering a human weight of 50 kg, a rat weight of 0.30 kg, and a human dose of 2 ng/kg/day, we obtained a rat dose of 7.18 ng/kg/day, which was rounded to 7.2 ng/kg/day of NDMA. In addition, NDMA toxicokinetics have been extensively characterized in rodents, showing rapid absorption, high oral bioavailability (> 90%), a half‐life of approximately 2–3 h, and acute oral LD_50_ values of ~40 mg/kg in rats and ~23 mg/kg in mice (IARC 2010; Lee et al. 1996; Verna et al. 1996; ATSDR 2022).

### 2.2 Animals

Wistar female (*n* = 22, 4 weeks, 230 ± 3.12 g) and male (*n* = 10, 4 weeks, 320 ± 2.89 g) rats were obtained from the Central Biotherium of São Paulo State University (UNESP). Animals were housed under controlled conditions: temperature of 22°C, 12 h light/12 h dark photoperiod, and 50% relative humidity, with ad libitum access to standard phytoestrogen‐free chow (Nuvilab CR1, Nuvital‐PR, Brazil) and water.

All procedures followed the Ethical Principles in Animal Research, as approved by the Ethics Committee for Animal Experimentation of the Institute of Biosciences, Botucatu/UNESP (Protocol: 9891060624).

### 2.3 Groups and Experimental Design

**FIGURE 1:**
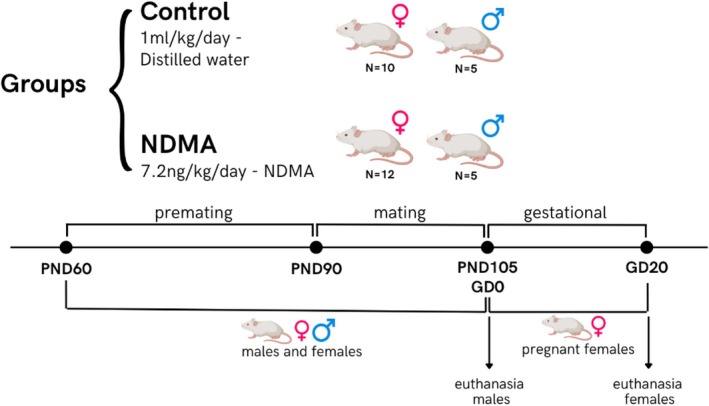
Experimental design.

Animals were weighed to confirm comparable average initial body weights between groups and then randomly assigned to two groups. The control group (5 males, 10 females) received vehicle only (deionized water, 1 mL/kg/day). The NDMA group (5 males, 12 females) received NDMA at 7.2 ng/kg/day via oral gavage. Males and females were exposed to NDMA or vehicle during three consecutive periods: preconception, mating, and gestation (Figure 1).

#### 2.3.1 Preconceptional Period

Both sexes were exposed from postnatal day (PND) 60 to 90 (31 days). *Mating period* (starting on PND 91): during this phase, vaginal lavages of the females were performed. Females in estrus phase were placed with a male sexually active from their respective group overnight, at the proportion of 1 male:1 female. In the next morning, the vaginal material was collected again. The presence of sperm in females and cornified cells (estrus) was considered gestational day 0 (GD 0). If pregnancy was not confirmed, the animals remained exposed for a maximum of 14 days, corresponding to the duration required for 3 to 4 complete estrous cycles. This period also allowed for approximately 3 to 4 mating attempts, extending up to PND 104, thereby maximizing the chances of successful fertilization (OECD 2016). After this period, the male rats were euthanized.

#### 2.3.2 Gestation

Upon confirmation of pregnancy, females continued exposure from GD 0 to GD 20 (21 days), allowing assessment of potential toxic effects during pre‐implantation, organogenesis, and fetal periods in accordance with OECD guidelines (OECD 2018).

During treatment, pregnant rats, maintained in individual cages, were weighed on alternate days to calculate the volume of NDMA to be administered, and clinical signs of toxicity (body weight, diarrhea, piloerection, bleeding, abnormal breathing, tremors, convulsions, and gear changes, posture, and reaction to manipulation) were investigated. In this study, adult male rats were used solely for mating. Their reproductive endpoints are being investigated in a separate study, specifically designed to assess the paternal impacts of NDMA.

### 2.4 Maternal Evaluations

On GD 20, pregnant females were anesthetized with thiopental sodium (50 mg/kg, i.p.) and euthanized. Blood samples were collected via cardiac puncture for hematological and biochemical analyses. Cesarean sections with hysterectomy and bilateral oophorectomy were performed. Ovaries were weighed, and corpora lutea were counted. The gravid uterus was removed intact and weighed. Fetuses and their placentas were individually excised. The following parameters were recorded: number of fetuses, fetal and placental weights, implantations, resorptions, and live/dead fetuses. These data were used to calculate reproductive indices: implantation efficiency, pre‐ and post‐implantation losses, and placental index (de Barros et al. 2016). Maternal organs (liver, kidneys, adrenal glands, spleen, and thyroid) were also excised and weighed.

#### 2.4.1 Biochemical and Hematological Parameters

Maternal blood samples were centrifuged at 1000 × g for 20 min at 4°C, and the resulting serum was analyzed for aspartate aminotransferase (AST), alanine aminotransferase (ALT), gamma‐glutamyltransferase (GGT), alkaline phosphatase (ALP), urea, creatinine, calcium, cholesterol, total protein, and albumin, which were quantified using Bioclin assay kits with a BioPlus 200 semi‐automated analyzer. Hematological parameters included total leukocytes, erythrocytes, platelets, hemoglobin concentration, and hematocrit; all parameters were evaluated on the SYXMEX semi‐automated analyzer. Blood smears were stained with the Rapid Panotic kit (Laborclin, Pinhais, PR, Brazil) for leukocyte differentiation, and 200 cells were classified into monocytes, eosinophils, basophils, neutrophils, and lymphocytes. Mean corpuscular volume (MCV), mean corpuscular hemoglobin (MCH), and mean corpuscular hemoglobin concentration (MCHC) were calculated.

#### 2.4.2 Histological Analysis

Placentas were initially fixed in 10% buffered formalin for 4 h, followed by immersion in 10% neutral buffered formalin (4% aqueous formaldehyde) for 24 h. Subsequently, tissues were embedded in Paraplast, sectioned at 5 μm using a rotary microtome, stained with hematoxylin and eosin (H&E), and examined under a light microscope. The thickness of the labyrinth and basal zones was measured, and any histological abnormalities were recorded. For each group, one placenta per litter was selected for analysis (*n* = 5 dams per group), and three sections were obtained and evaluated per placenta to ensure representative measurements.

#### 2.4.3 Placental Efficiency

Placental efficiency was assessed by calculating the fetal‐to‐placental weight ratio for each fetus (Fowden et al. 2009), an index reflecting maternal–fetal nutrient transfer and fetal resource utilization efficiency.

### 2.5 Fetal Development Evaluations

On GD 20, fetuses were classified as appropriate for gestational age (AGA), small for gestational age (SGA), or large for gestational age (LGA) based on body weight relative to gestational age, adapted from Soulimane‐Mokhtari et al. (2005). The classification thresholds were established using the distribution of fetal body weights in the control group: fetuses with weights below mean − (1.7 × SD) were classified as SGA, those within mean ± (1.7 × SD) as AGA, and those above mean + (1.7 × SD) as LGA.

#### 2.5.1 External Morphological Analysis of Fetuses

All fetuses were examined for external morphological abnormalities under a stereomicroscope, including assessment of ocular and cranial structures, limb development, neural tube closure, and the presence of hemorrhages, bruising, or other visible malformations.

#### 2.5.2 External Measurements

With the aid of a digital caliper, the craniocaudal distance (body length), the anogenital distance (AGD—from the genital tubercle to the anus), and the encephalic dimension (biparietal distance measured by the distance between the openings of the auditory canal) were measured. The relative AGD was obtained by calculating the ratio of the AGD to the cube root of the animal's weight.

#### 2.5.3 Visceral Parameters

Half of the fetuses from each litter were processed for visceral examination. After external assessment, fetuses were placed on a cold plate and subsequently fixed in Bouin's solution. Visceral analysis was performed using the method of Wilson (1965) for the head (five transverse sections) and the sectioning planes described by Barrow and Taylor (1969) for the thorax (three transverse sections). The following structures were evaluated for anomalies: cerebral ventricles, cornea, retina, inner ear, nasal cavity, palate, salivary glands, thyroid, esophagus, trachea, heart, thymus, liver, kidneys, bladder, ureters, and gonads. Morphological assessments were conducted according to Manson and Kang (1994), with adaptations by Oliveira et al. (2009).

#### 2.5.4 Skeletal Parameters

Selected fetuses were immersed in acetone for 24 h, followed by clearing in 0.8% KOH with the addition of saturated Alizarin Red solution. This procedure involved four solution changes at intervals of at least 24 h, according to the method of Staples and Schnell (1964). Ossification centers were evaluated based on the criteria established by Aliverti et al. (1979) to determine the degree of fetal skeletal development. Additional evaluations of anomalies and malformations followed Taylor (1986), covering cranial bones, sternum, clavicle, vertebrae, pelvis, and limb bones.

### 2.6 Statistical Analysis

Data are presented as mean ± standard error of the mean (SEM) for parametric data or median (interquartile range, Q1–Q3) for non‐parametric data, following normality assessment via the Shapiro–Wilk test. Intergroup comparisons were conducted using the unpaired *t*‐test for parametric data, with Welch's correction applied when appropriate, and the Mann–Whitney test for non‐parametric data. External fetal morphology, skeletal anomalies, and birth weight were analyzed using Fisher's exact test. A *p*‐value < 0.05 was considered statistically significant. Analyses were conducted using GraphPad InStat (version 8.02). All statistical analyses were conducted at the litter level, considering the dam as the experimental unit. Because both male and female fetuses were present in each litter, mixed‐effects models were applied with the dam included as a random effect to account for intra‐litter correlation and inter‐litter variability.

## 3 Results

### 3.1 Maternal Evaluations

There were no significant differences between groups in final body weight or weight gain during NDMA exposure (Table 1). Throughout the experimental period, none of the animals exhibited clinical or behavioral signs indicative of systemic or localized toxicity, nor was any evidence of miscarriage detected. Gross examination and maternal organ weights were comparable across all experimental groups (Table 1). Hematological analysis in the NDMA‐treated group showed a significant reduction in lymphocytes percentage, a significant increase in monocytes, and a decrease in basophil counts. Additionally, biochemical analysis also showed significantly elevated urea levels and reduced alanine aminotransferase (ALT) activity compared to the control group (Table 2). No significant differences were observed in the fertility parameters between the groups (Table 1). These results indicate that low‐dose NDMA exposure did not impair male reproductive success under the conditions of this study.

**TABLE 1:** Final body weight (g), relative organ weight (organ weight [g]/body weight × 100), and reproductive parameters of dams treated with NDMA.

	Control	NDMA	*p*
Final body weight (g)^a^	328.00 (320.50–354.90)	342.20 (312.20–355.00)	0.63
Relative organ weight (%)			
Liver	3.59 ± 60.84	3.73 ± 74.21	0.16
Kidney	0.27 ± 7.22	0.28 ± 5.86	0.30
Adrenal gland	1.21 ± 0.04	1.14 ± 0.05	0.39
Spleen	0.18 ± 6.17	0.18 ± 8.88	0.97
Ovary	3.47 ± 0.11	3.64 ± 0.12	0.31
Thyroid	0.43 ± 0.03	0.50 ± 0.06	0.34
Reproductive parameters			
Maternal weight gain (%)	32.27 ± 0.86	32.28 ± 1.26	0.54
Gravid uterus weight (g)	73.28 ± 3.47	63.27 ± 4.38	0.11
Number of live fetuses	12.60 ± 0.64	10.92 ± 0.74	0.11
Number of implantations^a^	13.50 (12.00–14.00)	11.50 (9.25–14.00)	0.21
Number of resorptions^a^	0 (0–1.25)	0 (0–1.00)	0.82
Number of corpora lutea^b^	14.30 ± 0.30	15.33 ± 1.09	0.38
Pre‐implantations loss (%)^a^	7.18 (0–15.00)	12.88 (0–47.38)	0.30
Post‐implantation loss (%)^a^	0 (0–0)	0 (0–8.90)	0.08
Sex ratio (M:F)	1.21 ± 0.20	1.37 ± 0.24	0.63
Fertility potential^a^	92.82 (85.00–100.00)	87.12 (52.62–100.00)	0.30

*Note:* Values are expressed as mean ± SEM. Unpaired *t*‐test. *p* > 0.05. Unpaired *t*‐test with Welch's correction. *p* > 0.05. *n* = 10 control and 12 NDMA‐treated dams.

^a^ Values are expressed as median (Q1–Q3). Mann–Whitney test. *p* > 0.05.

^b^ Values are expressed as mean ± SEM.

**TABLE 2:** Hematological and biochemical parameters of the dams treated with NDMA.

	Control	NDMA	*p*
Hematological			
Hematocrit (%)	34.08 ± 0.59	35.41 ± 0.77	0.20
Hemoglobin (g/dL)	11.66 ± 0.17	11.97 ± 0.24	0.31
Erythrocytes (10^6^/μL)^a^	5.89 (5.82–6.25)	6.14 (5.81–6.66)	0.42
MCV (fL)	56.44 ± 0.36	56.47 ± 0.36	0.95
MCH (pg)	19.30 ± 0.14	19.11 ± 0.19	0.45
MCHC (%)	34.21 ± 0.20	33.82 ± 0.21	0.19
Leukocytes (10^3^/μL)	7.61 ± 0.55	6.61 ± 0.31	0.11
Lymphocytes (%)^a^	74.00 (65.00–75.50)	61.00 (54.00–64.00)	0.0004***
Neutrophils (%)	16.44 ± 1.76	20.64 ± 1.48	0.08
Monocytes (%)^b^	8.22 ± 0.86	17.00 ± 1.62	0.0002***
Eosinophils (%)^a^	1.00 (0.50–2.50)	1.00 (0–1.00)	0.39
Basophils (%)^a^	2.00 (1.50–3.00)	1.00 (0–3.00)	0.04*
Platelets (10^3^/μL)^a^	861.00 (610.00–919.00)	868.00 (838.00–901.00)	0.84
Biochemical			
Total protein (g/dL)	5.22 ± 0.13	5.56 ± 0.25	0.26
Albumin (g/dL)	2.42 ± 0.06	2.63 ± 0.09	0.09
Creatinine (mg/dL)^b^	0.50 ± 0.00	0.49 ± 0.03	0.76
Urea (mg/dL)^b^	45.89 ± 1.81	59.82 ± 5.27	0.03*
AST (U/L)^b^	118.70 ± 13.51	75.60 ± 3.67	0.01*
ALT (U/L)^b^	75.33 ± 5.74	67.64 ± 7.04	0.42
GAMMA GT (U/L)^a^	0 (0–1.50)	0 (0–0)	0.31
Alkaline Phosphatase (U/L)	183.00 ± 13.77	190.80 ± 13.35	0.70
Cholesterol	67.78 ± 3.75	82.18 ± 6.62	0.09
Calcium^a^	17.80 (16.40–25.95)	18.60 (18.10–26.60)	0.54
Uric acid^a^	8.10 (5.70–10.50)	5.25 (4.63–8.95)	0.27

*Note:* Values are expressed as mean ± SEM. Unpaired *t*‐test.

Abbreviations: ALT, Alanine aminotransferase; AST, Aspartate aminotransferase; GAMMA GT, Gamma glutamyl transferase; MCH, mean corpuscular hemoglobin; MCHC, mean corpuscular hemoglobin concentration; MCV, mean corpuscular volume; *n* = 10 control and 12 NDMA‐treated dams.

* *p* < 0.05.

*** *p* < 0.001.

^a^ Values are expressed as median (Q1–Q3). Mann–Whitney test.

^b^ Values are expressed as mean ± SEM. Unpaired *t*‐test with Welch's correction.

### 3.2 Placental Evaluations

**FIGURE 2:**
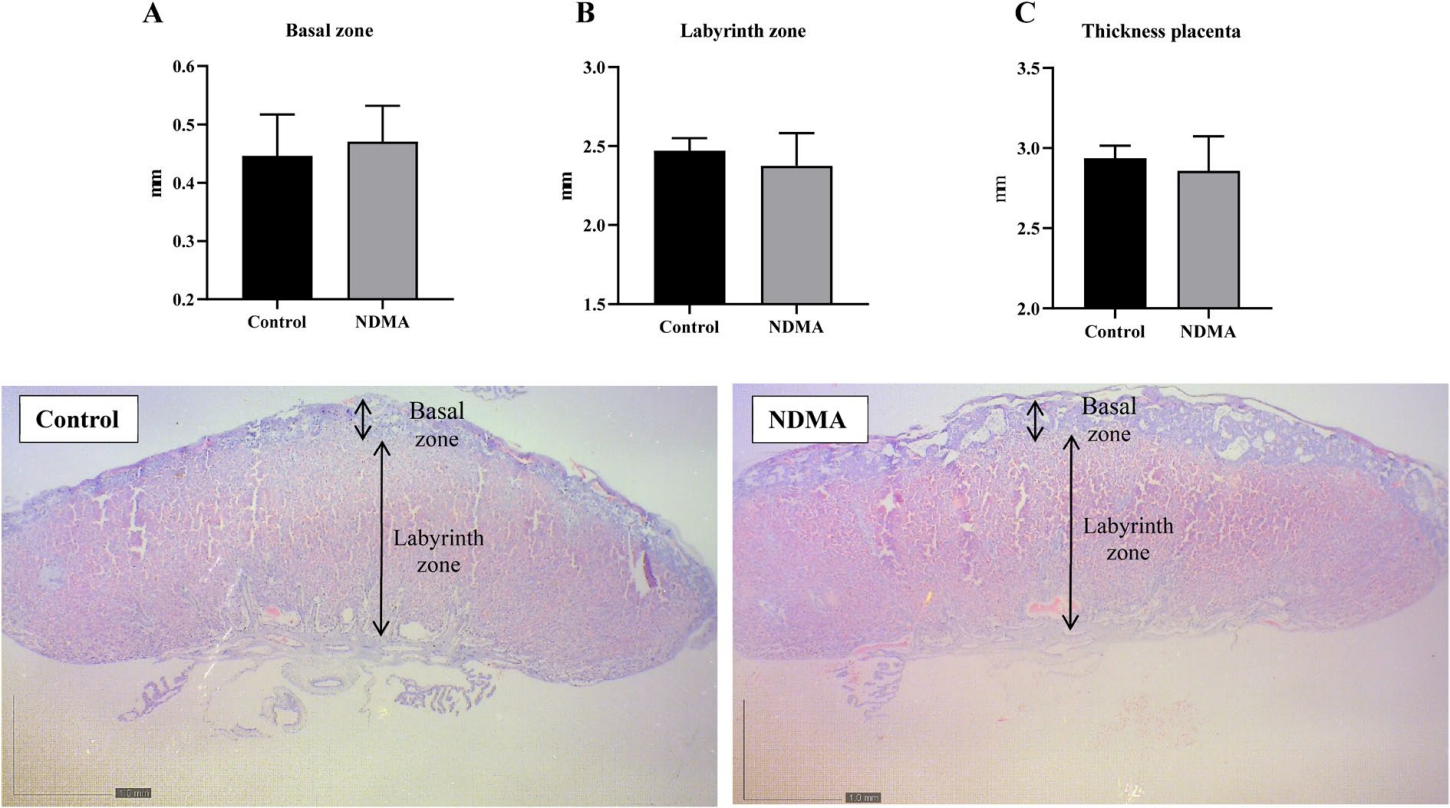
Measurement of heights of the Basal zone (A), Labyrinth zone (B), and the total placental thickness (C). Photomicrographs of placentas at low magnification (0.8) from the control and NDMA groups. Values expressed as median (Q1–Q3). *n* = 5 dams (1 placenta per litter)/group. Mann–Whitney test, *p* > 0.05.

Histological analysis of the placenta (Figure 2) showed no significant differences between groups in the thickness of the basal and labyrinth zones. Placental weight associated with male fetuses showed a slight, non‐significant reduction (*p* = 0.07), along with a borderline increase in placental efficiency (*p* = 0.05). Both placental weight and placental efficiency in females remained within normal physiological ranges across all groups (Table 3).

**TABLE 3:** Embryofetal parameters of fetuses treated with NDMA.

	Control	NDMA	*p*
*Male fetuses*	(*n* = 65)	(*n* = 70)	
Body weight (g)	4.06 ± 0.04	4.14 ± 0.04	0.14
Placenta weight (g)	0.54 ± 0.01	0.51 ± 0.01	0.07
Placenta efficiency	7.73 ± 0.14	8.10 ± 0.13	0.05
Relative AGD	2.90 ± 0.02	2.89 ± 0.02	0.80
Craniocaudal length (mm)	54.99 ± 0.25	55.38 ± 0.21	0.23
Head measurement (mm)^a^	9.83 (9.50–10.06)	9.82 (9.52–10.07)	0.94
*Female fetuses*	(*n* = 61)	(*n* = 62)	
Body weight (g)^a^	3.84 (3.64–4.08)	3.91 (3.74–4.09)	0.46
Placenta weight (g)^a^	0.48 (0.43–0.53)	0.49 (0.45–0.51)	0.82
Placenta efficiency	8.01 ± 0.15	8.12 ± 0.14	0.60
Relative AGD	1.84 ± 0.02	1.85 ± 0.01	0.97
Craniocaudal length (mm)^a^	54.77 (53.82–55.97)	55.17 (53.77–55.99)	0.81
Head measurement (mm)	9.72 ± 0.04	9.67 ± 0.05	0.44

*Note:* Values are expressed as mean ± SEM. Unpaired *t*‐test. *p* > 0.05. The litter was used as the statistical unit of comparison. *n* = 10–12 litters/group. Mann–Whitney test. *p* > 0.05.

Abbreviation: AGD, anogenital distance.

^a^ Values are expressed as median (Q1–Q3).

### 3.3 Fetal Evaluation

**FIGURE 3:**
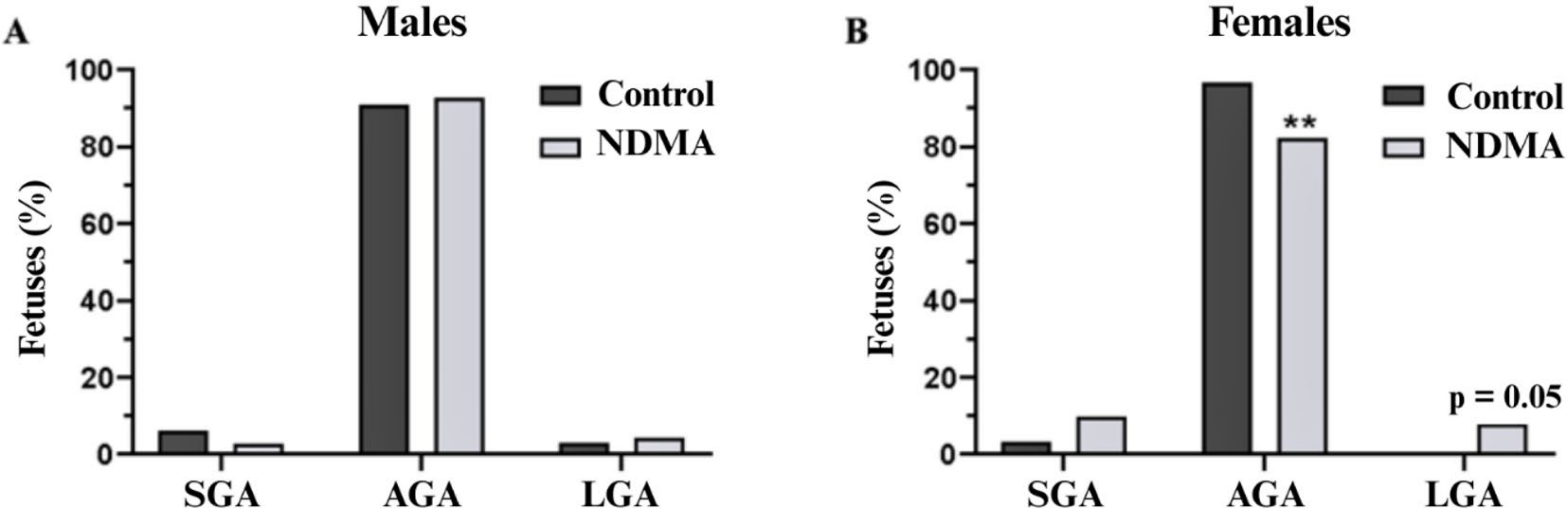
Distribution of male (A) and female (B) fetal weights on GD 20. Control group: Males (*n* = 65), females (*n* = 61); NDMA group: Males (*n* = 70), females (*n* = 62). Classification of SGA, AGA, and LGA was based on body weight relative to gestational age, using thresholds defined as mean ± (1.7 × SD) of the control group. Values are expressed as percentages. Fisher's test. **p < 0.01. AGA, appropriate for gestational age; LGA, large for gestational age; SGA, small for gestational age.

Although no statistically significant differences were observed between the groups in mean fetal weight, craniocaudal length, or encephalic measurements (Table 3), a significant reduction in fetal weight was detected in females classified as adequate for gestational age (AGA) and a proportional increase in large for gestational age (LGA) in the NMDA group (Figure 3).

**FIGURE 4:**
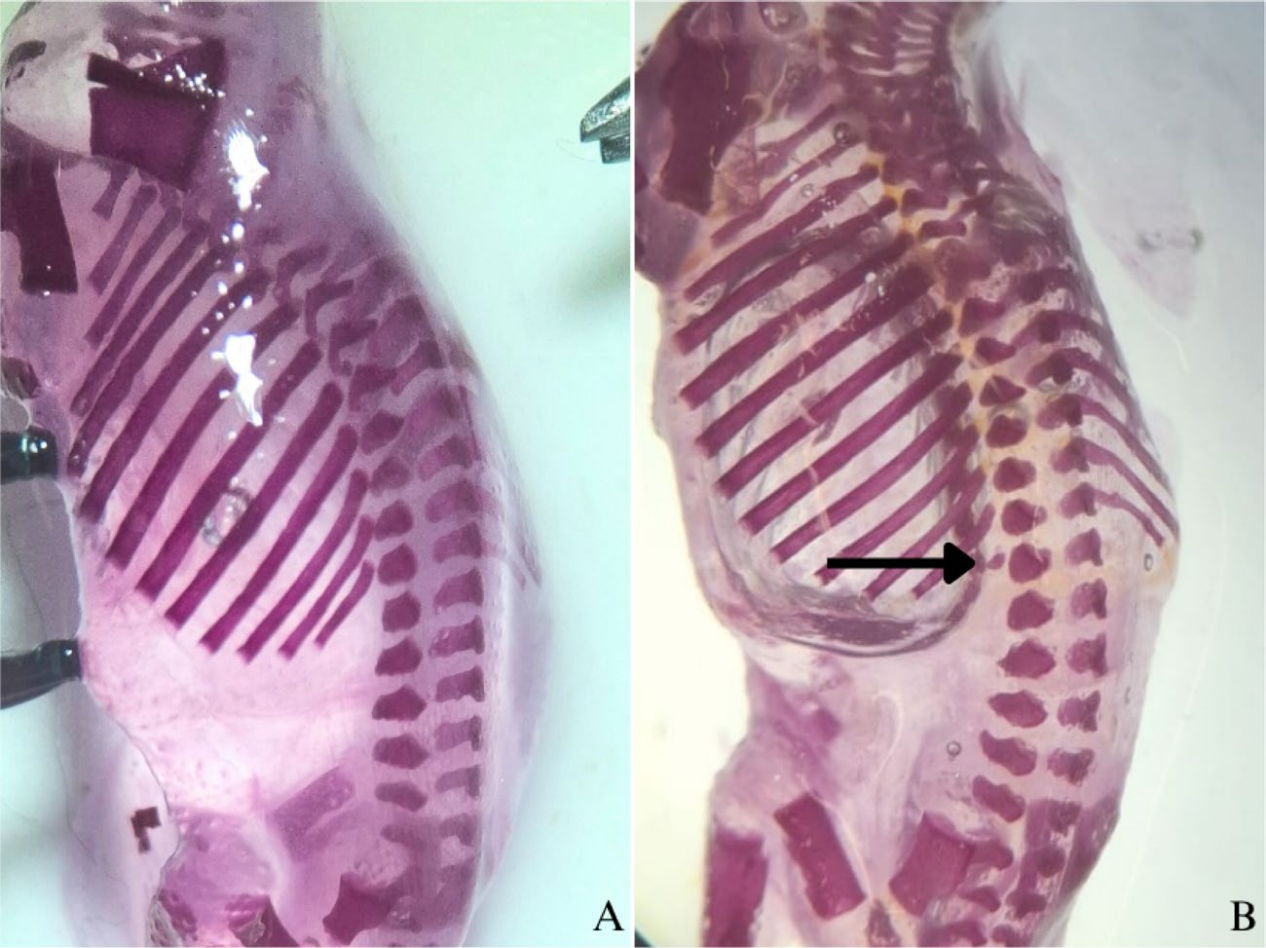
Supernumerary rib in fetuses from the NDMA group (B), indicated by the arrow. No supernumerary rib is observed in the control group (A).

No significant changes were detected in parameters related to external morphology or visceral structures (presence/absence of enlargement or atrophy of the cerebral ventricles, cornea, retina, inner ear, nasal cavity, palate, salivary gland, thyroid, esophagus, trachea, heart, thymus, liver, kidneys, bladder, ureters, and gonads) (Table 4). However, skeletal analysis (Table 4) revealed an increase in the percentage of supernumerary ribs in fetuses from the NDMA‐treated group compared to the control group (Figures 3 and 4).

**TABLE 4:** Skeletal and visceral evaluations of the fetuses treated with NDMA.

	Control (*n* = 63)	NDMA (*n* = 63)	*p*
Skeletal			
Supernumerary ribs	26.98	49.20	0.016*
Sternal anomalies	69.84	65.08	0.70
Incomplete ossification of the skull	20.63	19.05	1.00
Spinal and hip anomalies	9.52	6.34	0.74
Visceral			
Brain anomalies	0	4.28	0.10
Kidney anomalies	2.85	0	1.0
Eye anomalies	1.42	0	0.50
Organ size anomalies	0	4.28	0.10
Heart anomalies	0	0	1.0
Lung anomalies	0	0	1.0
Trachea anomalies	0	0	1.0
Liver anomalies	0	0	1.0

*Note:* Values are expressed as percentages. The litter was used as the statistical unit of comparison. *n* = 10–12 litters/group. Fisher's test.

* *p* < 0.05.

## 4 Discussion

While previous studies have primarily focused on maternal exposure, the present study is the first to investigate the combined effects of both maternal and paternal preconception exposure to environmentally relevant doses of NDMA—doses considered safe by regulatory agencies (FDA 2021)—on maternal health, placental morphology, and embryofetal development in a rat model. This dual‐exposure approach more accurately reflects potential human scenarios, in which both parents may be simultaneously exposed to environmental contaminants, potentially affecting reproductive and developmental outcomes (Liu et al. 2025).

Our findings demonstrate that NDMA exposure, at the dose and duration used in this study, caused two major effects: hematological and biochemical alterations in pregnant females and skeletal anomalies in their offspring. Previous animal studies have typically employed much higher doses of NDMA to assess its reproductive and developmental toxicity. For example, one study reported that single oral doses of NDMA (15–20 mg/kg) caused acute toxicity in pregnant rats, particularly in late gestation. When administered on GD 13 or 18, NDMA increased mitotic activity in the liver and adrenal glands, altered liver enzyme activity, reduced serum protein levels, and decreased fetal weight (Nishie 1983). Similarly, in Sprague Dawley rats, doses ranging from 250 to 1000 mg/kg/day administered by gavage during gestation induced fetal developmental changes, including skeletal variations, without significant maternal toxicity (NTP 2020). In contrast, our study employed substantially lower, environmentally relevant doses, with both parents exposed.

Consistent with prior reports indicating NDMA's immunomodulatory effects (ATSDR 2022; Gómez et al. 1988), we observed a significant reduction in lymphocyte percentages and an increase in monocyte counts in NDMA‐exposed females. These hematological alterations may signal systemic inflammatory responses or physiological stress secondary to NDMA metabolism and the consequent generation of reactive intermediates (ATSDR 2022). Supporting this, previous studies have demonstrated that prenatal NDMA exposure induces oxidative stress and apoptosis in fetal tissues, contributing to developmental toxicity (Zhou et al. 2022). Additionally, chronic low‐dose NDMA exposure has been shown to impair immune function and reproductive health in rats, corroborating our observations (Wang et al. 2021). Moreover, the observed elevation in urea levels, coupled with reduced ALT levels, suggests potential renal involvement and mild hepatocellular compromise, despite the absence of overt toxicity in organ weights or gross pathology. These findings are consistent with similar biochemical alterations reported in rodents subjected to subchronic NDMA exposure (Johnson et al. 2021; Peto et al. 1984).

Despite these maternal alterations, fertility potential and key embryo‐fetal developmental parameters—including fetal viability, craniocaudal length, and encephalic measurements—remained unaffected. These findings corroborate previous evidence suggesting that NDMA is not overtly teratogenic at low doses (Anderson et al. 1989; Huang and Catalano 1994). However, a significant reduction in fetal weight—an established indicator of potential developmental perturbations (Sandovici et al. 2012)—was observed among female fetuses classified as AGA within the NDMA‐exposed group. This was accompanied by an increase in the proportion of fetuses classified as LGA in the NDMA group, reaching the threshold for statistical significance (*p* = 0.05), which may reflect a disturbance in intrauterine growth regulation. This sex‐specific response may reflect differential susceptibility to environmental toxicants, as previously reported in developmental toxicology studies (Gore et al. 2015; Ho et al. 2017). Females are generally considered more sensitive to toxic insults during development, as recognized by the OECD guidelines for developmental and reproductive toxicity testing (OECD 2018). Mechanistic studies have proposed that NDMA‐induced reproductive toxicity may involve oxidative stress, epigenetic modifications, and endocrine disruption (Pang et al. 2023), which could underlie the observed sex‐dependent vulnerability. Supporting this, Liu et al. (2025) emphasize nitrosamines' ability to disrupt gametogenesis and cause heritable reproductive damage, reinforcing the need to examine preconception exposures.

The placenta, a key maternal‐fetal interface, mediates essential processes during gestation, particularly nutrient transport and energy storage (Akison et al. 2017). Previous research has demonstrated that maternal NDMA exposure can disrupt placental development and impair fetal growth in murine models (Li et al. 2020). However, in our study, placental morphology—including the dimensions of the basal and labyrinth zones, as well as placental weight and efficiency—remained within normal physiological ranges. These findings suggest that any placental functional alterations potentially induced by NDMA exposure may be subtle or not detectable through standard histomorphometric assessments. Interestingly, the placentas of the male fetuses showed a slight difference between the groups, with a decrease in placental weight and an increase in placental efficiency, even though the fetal weight of the males was unchanged. Any change in these parameters could indicate that this organ may have undergone some challenge during gestation (Fowden et al. 2009), which, in our study, appears to have been induced by NDMA exposure. Further investigations employing molecular or functional assays are warranted to elucidate potential placental adaptations or dysfunctions in response to NDMA exposure.

Skeletal variation is widely recognized as a marker of developmental perturbation and has been associated with in utero exposure to various environmental toxicants (Carney et al. 2004; Fleeman and Saunders 2011). Delayed ossification of the sternum and the presence of supernumerary ribs are considered reliable indicators of treatment‐induced fetal growth retardation (Chahoud and Paumgartten 2005). In our study, NDMA‐exposed fetuses exhibited skeletal alterations, with a particularly elevated occurrence of supernumerary ribs. While a baseline occurrence of skeletal anomalies is considered physiologically normal across mammalian species (Chernoff and Rogers 2004), the significantly elevated incidence of supernumerary ribs in the NDMA‐exposed group may suggest perturbations in somitogenesis or axial skeletal patterning. Such defects are plausibly linked to NDMA‐induced oxidative stress and genotoxicity, which have been previously implicated in the disruption of embryonic developmental pathways (ATSDR 2022). However, no significant external or visceral malformations were identified, indicating that NDMA at this exposure level does not elicit gross teratogenic effects in rats. This is consistent with previous evidence indicating that NDMA‐induced embryotoxicity primarily occurs at high doses or in conjunction with maternal toxicity (Nishie 1983; Anderson et al. 1989; Huang and Catalano 1994).

A limitation of our study is that the design does not allow a clear separation of paternal and maternal contributions to the observed offspring outcomes, since both parents were exposed prior to conception and females remained exposed throughout gestation. Thus, maternal hematological and biochemical alterations at GD20 are most likely attributable to gestational exposure, whereas fetal endpoints should be interpreted as the result of combined paternal and maternal influences. To address this limitation, ongoing studies in our group are specifically evaluating the paternal effects of NDMA, including sperm analysis and testicular endpoints. Future studies should use separate parental exposure groups to clarify whether the observed effects are attributable to maternal, paternal, or combined influences.

In conclusion, these findings provide novel insights into the developmental and reproductive toxicity profile of NDMA at doses approaching regulatory limits, with a unique focus on combined parental exposure. While overt teratogenicity was not observed, the hematological and biochemical alterations in dams, reduction in female fetal weight, and increased skeletal variations highlight the potential for adverse effects even at low exposure levels. These results underscore the importance of continued toxicological evaluations of NDMA, particularly regarding chronic, low‐dose exposures that more accurately reflect typical human environmental contact. Future studies should elucidate the mechanistic pathways underlying the observed sex‐specific fetal growth reduction and skeletal anomalies, as well as assess the potential long‐term postnatal consequences of prenatal NDMA exposure.

## Conflicts of Interest

The authors declare no conflicts of interest.

## Data Availability

The data that support the findings of this study are available from the corresponding author upon reasonable request.
